# Using spectral and temporal filters with EEG signal to predict the temporal lobe epilepsy outcome after antiseizure medication via machine learning

**DOI:** 10.1038/s41598-023-49255-2

**Published:** 2023-12-18

**Authors:** Youmin Shin, Sungeun Hwang, Seung-Bo Lee, Hyoshin Son, Kon Chu, Ki-Young Jung, Sang Kun Lee, Kyung-Il Park, Young-Gon Kim

**Affiliations:** 1 Department of Transdisciplinary Medicine, Seoul National University Hospital, Seoul, Republic of Korea; 2 Interdisciplinary Program in Bio-Engineering, Seoul National University, Seoul, Korea; 3 Department of Neurology, Ewha Womans University Mokdong Hospital, Seoul, Republic of Korea; 4 Department of Medical Informatics, Keimyung University School of Medicine, Daegu, Republic of Korea; 5 Department of Neurosurgery, Seoul National University Hospital, Seoul, Republic of Korea; 6 Department of Neurology, Seoul National University Hospital, Seoul, Republic of Korea; 7 Department of Neurology, Seoul National University College of Medicine, Seoul, Republic of Korea; 8 Department of Neurology, Seoul National University Hospital Healthcare System Gangnam Center, Seoul, Republic of Korea; 9 Department of Medicine, Seoul National University College of Medicine, Seoul, Korea

**Keywords:** Computational neuroscience, Machine learning, Neurological disorders

## Abstract

Epilepsy is a neurological disorder in which the brain is transiently altered**.** Predicting outcomes in epilepsy is essential for providing feedback that can foster improved outcomes in the future. This study aimed to investigate whether applying spectral and temporal filters to resting-state electroencephalography (EEG) signals could improve the prediction of outcomes for patients taking antiseizure medication to treat temporal lobe epilepsy (TLE). We collected EEG data from a total of 46 patients (divided into a seizure-free group (SF, n = 22) and a non-seizure-free group (NSF, n = 24)) with TLE and retrospectively reviewed their clinical data. We segmented spectral and temporal ranges with various time-domain features (Hjorth parameters, statistical parameters, energy, zero-crossing rate, inter-channel correlation, inter-channel phase locking value and spectral information derived from Fourier transform, Stockwell transform, and wavelet transform) and compared their performance by applying an optimal frequency strategy, an optimal duration strategy, and a combination strategy. For all time-domain features, the optimal frequency and time combination strategy showed the highest performance in distinguishing SF patients from NSF patients (area under the curve (AUC) = 0.790 ± 0.159). Furthermore, optimal performance was achieved by utilizing a feature vector derived from statistical parameters within the 39- to 41-Hz frequency band with a window length of 210 s, as evidenced by an AUC of 0.748. By identifying the optimal parameters, we improved the performance of the prediction model. These parameters can serve as standard parameters for predicting outcomes based on resting-state EEG signals.

## Introduction

Epilepsy is a neurological disorder in which transient alteration of brain functions due to hyperexcitable neurons and their network causes seizures^1,2^. Patients with epilepsy suffer from recurrent paroxysmal symptoms, such as loss of consciousness, tonic‒clonic seizures, and behavioral changes^3^, as well as persistent comorbidities, such as depression and anxiety. Thus, epilepsy is a highly burdensome disease. The causes of epilepsy include nutritional status during pregnancy, complications during childbirth, head injury, exposure to toxic substances, brain infections, tumors, strokes, and degenerative changes in the brain^4–6^.

Antiseizure medication (ASM) is an initial option and the mainstay in epilepsy treatment^7^. While more than 30 ASMs have been developed and utilized^8^, approximately one-third of epilepsy patients are still medically refractory^9–11^. Among medically refractory patients, surgically remediable patients undergo resection of the seizure focus area. The International League Against Epilepsy (ILAE) defines medically refractory epilepsy as the persistence of seizures even after adequate trials of two ASMs^12^. However, given the paucity of information regarding seizures induced by cognitive problems, nocturnal seizures, and amnesia after seizures^13^, more objective and commonly utilized tools (such as EEG) are needed. Although EEG is used to diagnose and assess clinical status^14^, conventional visual readings of EEG recordings might be insufficient for evaluating brain status, especially for determining future outcomes.

More than 60% of newly diagnosed epilepsy patients enter long-term remission; therefore, predicting the outcome of epilepsy is crucial not only for patients but also for their families^10^. Additionally, predicting outcomes in epilepsy is essential for providing feedback that can lead to improved outcomes^15^.

Accordingly, many recent machine learning (ML) studies have used resting-state EEG data to predict the long-term outcomes (i.e., seizure-free (SF) or non-seizure-free (NSF)) of ASM treatment. These data can be used as a tool to characterize the neurophysiological effects of ASMs in epilepsy patients. Zhang et al.^16^ proposed a prediction model that used sample entropy and a support vector machine (SVM) to predict the attainment of SF status in epilepsy patients treated with levetiracetam (LEV). Croce et al.^17^ used a partial least square regression with standard deviation as a feature. Ricci et al.^18^ employed the power spectral density and phase locking value to measure brain activity and connectivity. These three studies were conducted among epilepsy patients who received LEV as their first ASM. Additionally, Lanzone et al.^19^ examined the effect of perampanel in patients with focal epilepsy. Notably, all these studies focused on spectral analysis of the delta (0.5–4 Hz), theta (4–7.5 Hz), alpha (7.5–12 Hz), beta (12–30 Hz), and gamma (30–60 Hz) bands; this method is called pharmaco-EEG^20^ and involves the quantitative analysis of the EEG data to document the effect of drugs. However, more specific frequency bands should be investigated to optimize the predictive performance of the model^21^. In addition to spectral analysis, temporal analysis should also be performed^22–24^. Moreover, the optimal features should be explored^16^. From a clinical perspective, the prediction of outcomes should be performed for individuals undergoing both polytherapy and monotherapy, as polytherapy users account for approximately one-third of all epilepsy patients^25^. Furthermore, predictions in the context of monotherapy, especially for one type of drug, do not reflect real-world practice.

In the present study, we adopted ML classifiers to predict patient outcomes and to identify the optimal spectral and temporal features. We implemented the random forest (RF)^26^ model in the main experiment, while linear discriminant analysis (LDA)^27^, XGBoost (XGB)^28^, and CatBOFTSoost (CATB)^29^ were used for the additional experiment.

The goal of this study was to use resting-state EEG signals to determine whether spectral and temporal filters can be used to predict the outcomes of patients with temporal lobe epilepsy (TLE), which is the most common type of focal epilepsy. Four different analysis strategies were designed with various time-domain features: the optimal frequency strategy (OFS), optimal time strategy (OTS), optimal frequency and time strategy (OFTS), and no strategy (NS). Finally, some interpretations of the experimental results were provided based on established theories.

## Results

### Comparison of the effects of the analysis strategies

**Figure 1:**
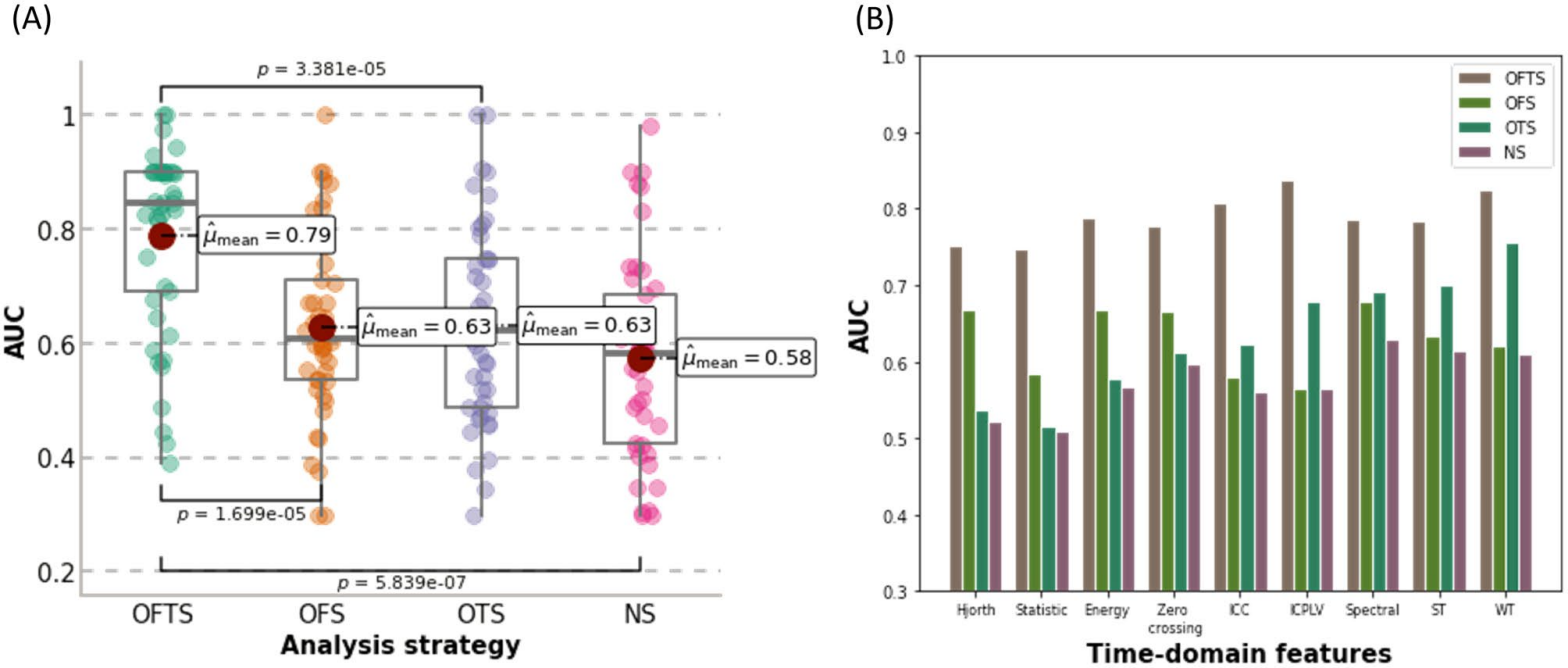
Comparative Analysis of AUC Values Across Different Analysis Strategies and Features. (**A**) Boxplot representation showing the aggregate AUC values for each analysis strategy. This part of the figure combines results from distinct nine feature groups, with each strategy displaying 45 data points. These points represent the AUC values obtained from fivefold cross-validation for each of the feature groups, thereby illustrating the collective performance across multiple validation scenarios. (**B**) Bar charts depicting the AUC values for each individual feature under the different analysis strategies. The chart provides a feature-specific comparison, illustrating how each feature group contributes to the overall efficacy of the strategies.

The effect of each analysis strategy was compared based on the classification performance of all feature groups combined (Fig. 1A) and each feature group separately (Fig. 1B). The box plot of the area under the receiver operating characteristic curve (AUC) values for each analysis strategy shows that the OFTS strategy had significantly higher AUC values (0.790 ± 0.159) than the OTS strategy (0.631 ± 0.170, $$p_{OFTS - OTS} \; < \;0.001,\;{{ {\text Cliff}^{\prime}s }}\;{\text{delta}}_{OFTS - OTS} \; = \;0.526)$$, the OFS strategy (0.629 ± 0.160, $$p_{OFTS - OFS} \; < \;0.001,{ }\;{{{\text Cliff}^{\prime}s }}\;{\text{delta}}_{OFTS - OFS} \; = \;0.507$$) and NS (0.575 ± 0.174, $$p_{OFTS - NS} \; < \;0.001,{ }\;{{{\text  Cliff}^{\prime}s}}\;{\text{ delta}}_{OFTS - NS} \; = \;0.611$$$$)$$. The bar charts show that the AUC of each feature group under the OFTS strategy was higher than that under the other strategies.

### Comparison of the performance of time-domain features

The classification performance of each time-domain feature under the OFTS strategy is presented in Table 1. Feature group F yielded the best AUC (0.838 ± 0.204), and Feature group B yielded the best accuracy (ACC; 0.824 ± 0.135), as shown in Table 1. Since Feature group B showed the highest performance on all metrics except for the AUC, Feature group B was evaluated with various ML classifiers. In this experiment, XGB showed the highest performance (AUC: 0.765 ± 0.179, ACC: 0.827 ± 0.112) on all metrics except for the true negative rate (TNR), positive predictive value (PPV), and negative predictive value (NPV). Detailed information about these analyses is provided in Supplementary Table 1.

**Table 1:** The classification performance of each time-domain feature under the optimal frequency and time strategy.

	AUC	ACC	F1 score	TPR	TNR	PPV	NPV
Feature group A	0.751 ± 0.097	0.668 ± 0.111	0.706 ± 0.086	0.658 ± 0.091	0.706 ± 0.135	0.691 ± 0.030	0.647 ± 0.034
Feature group B	0.748 ± 0.163	**0.824 ± 0.135**	**0.867 ± 0.076**	**0.801 ± 0.020**	**0.794 ± 0.071**	**0.800 ± 0.076**	**0.801 ± 0.158**
Feature group C	0.789 ± 0.185	0.656 ± 0.089	0.644 ± 0.091	0.658 ± 0.135	0.665 ± 0.030	0.654 ± 0.131	0.644 ± 0.157
Feature group D	0.778 ± 0.162	0.646 ± 0.147	0.658 ± 0.091	0.665 ± 0.156	0.647 ± 0.034	0.644 ± 0.104	0.633 ± 0.184
Feature group E	0.808 ± 0.212	0.783 ± 0.186	0.766 ± 0.109	0.748 ± 0.051	0.761 ± 0.104	0.738 ± 0.051	0.748 ± 0.073
Feature group F	0.838 ± 0.048	0.734 ± 0.093	0.748 ± 0.036	0.723 ± 0.131	0.718 ± 0.157	0.748 ± 0.073	0.766 ± 0.109
Feature group G	0.786 ± 0.204	0.773 ± 0.120	0.766 ± 0.109	0.738 ± 0.051	0.748 ± 0.073	0.766 ± 0.109	0.766 ± 0.109
Feature group H	0.784 ± 0.152	0.643 ± 0.133	0.633 ± 0.184	0.654 ± 0.034	0.692 ± 0.261	0.654 ± 0.034	0.658 ± 0.123
Feature group H	**0.825 ± 0.143**	0.757 ± 0.101	0.748 ± 0.073	0.766 ± 0.109	0.753 ± 0.036	0.761 ± 0.104	0.766 ± 0.020

Significant values are in [bold].

The optimal frequency band and window length for each feature were as follows. Values in bold represent the best results within each column. Feature group A: frequency of 1–3 Hz, length of 28 s; Feature group B: frequency of 39–41 Hz, length of 210 s; Feature group C: frequency of 3–5 Hz, length of 270 s; Feature group D: frequency of 42–44 Hz, length of 14 s; Feature group E: frequency of 22–24 Hz, length of 270 s; Feature group F: frequency of 37–39 Hz, length of 300 s; Feature group G: frequency of 27–29 Hz, length of 150 s; Feature group H: frequency of 31–33 Hz, length of 150 s and Feature group I: frequency of 36–38 Hz, length of 180 s.

AUC, area under the receiver-operating characteristic curve; ACC, accuracy; TPR, true positive rate; TNR, true negative rate; PPV, positive predictive value; NPV, negative predictive value.

### Comparison of the major feature values between SF and NSF patients

**Figure 2:**
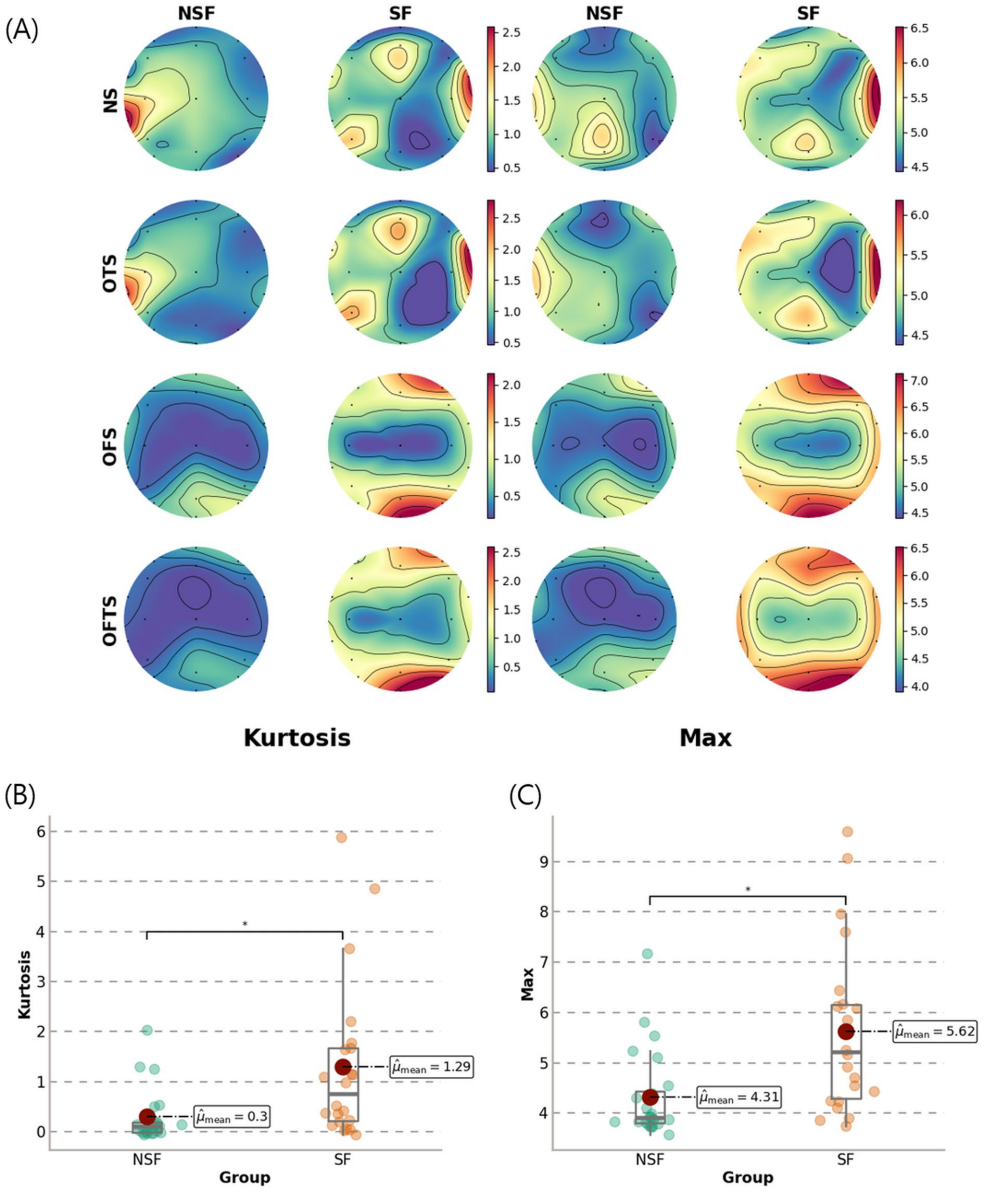
(**A**) Topology plots for each analysis strategy using the kurtosis and maximum value of Feature 2. Each topology plot consisted of the average value of each group (seizure-free (SF) or non-seizure-free (NSF)). (**B**, **C**) Comparison of SF vs. NSF groups according to the kurtosis (**B**) and the maximum value (**C**) under OFTS for each patient. For each patient's kurtosis and maximum values in (**B**, **C**), the average for all EEG channels was used. Asterisks indicate that the p value is less than 0.05.

Figure 2A shows topology plots demonstrating the ability of Feature group B (statistical parameters) to distinguish between the SF and NSF groups. The kurtosis and maximum value were extracted from the EEG signals of all TLE patients (SF group: 22 patients, NSF group: 24 patients), and the EEG channel-wise average of patients was used to obtain the kurtosis and maximum value. Among the statistical parameters for Feature group B, the kurtosis and maximum value were selected since the values showed significant differences between the SF and NSF groups ($p_{kurtosis} = 0.002$, ${{{\text Cliff}^{\prime}s delta}}_{Kurtosis} = 0.576$, $p_{max} < 0.001,{ }\;\;{{ {\text Cliff}^{\prime}s delta}}_{max} = 0.570$) (Fig. 2B, C). The patterns of the topology plots were compared quantitatively using cosine similarity (CS)^30^ and Euclidean distance (ED)^31^. For kurtosis, compared with NS, the OTS strategy showed a slight increase in CS (${\text{CS}}_{OTS - NS} = 0.013$) and ED ($ED_{OTS - NS} = 0.342$), but the OFS strategy showed an increase in CS (${\text{CS}}_{OFS - NS} = 0.169$) and a decrease in ED (${\text{ED}}_{OFS - NS} = - \;0.023$). The OFTS strategy yielded the highest ED (4.929), with a CS (0.936) close to 1. At the maximum value, all strategies yielded CS values close to 1. Furthermore, ED was lower under the OTS strategy ($ED_{OTS - NS} = - \;0.212$) and higher under the OFS strategy ($ED_{OFS - NS} = 2.199$) than under NS. The OFTS strategy yielded the highest ED (5.935), similar to the findings for kurtosis (Supplementary Table 2).

### Optimal EEG window length and optimal frequency band of the EEG signals for SF prediction

**Figure 3:**
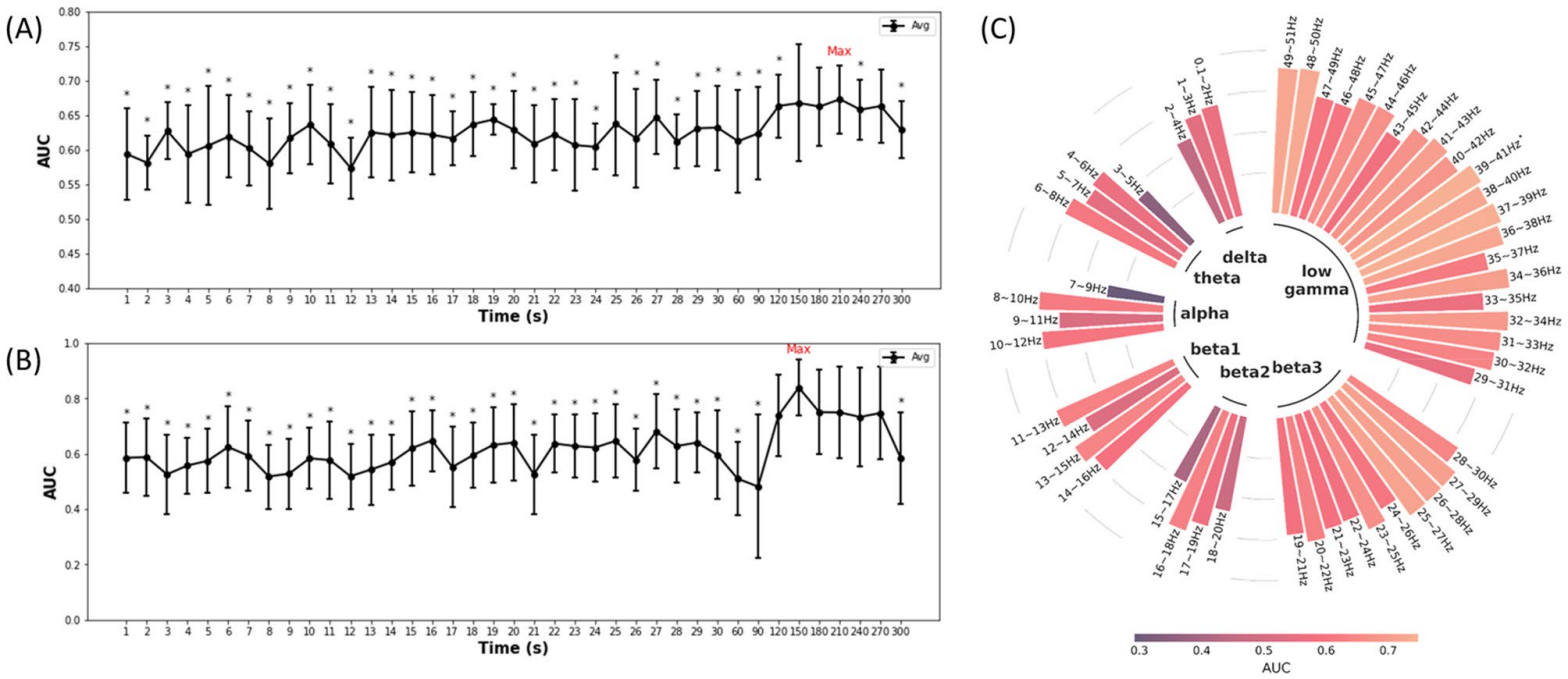
(**A**) Average performance (AUC) for all feature groups with each EEG window length on the optimal frequency band. Asterisks indicate that the p value is lower than 0.05 compared with the AUC at 270 s. (**B**) Performance (AUC) of Feature 2 on the optimal frequency band at each EEG window length. Asterisks indicate that the p value is lower than 0.05 compared with the AUC at 150 s. (**C**) Performance (AUC) of Feature 2 at the optimal EEG window length for each frequency band. The frequency range of each band is as follows: delta band, 0.1–4 Hz; theta band, 4–8 Hz; alpha band, 8–12 Hz; beta1 (low beta) band, 12–16 Hz; beta2 (beta) band, 16–20 Hz; beta3 (high beta) band, 20–30 Hz; and low gamma band, 30–50 Hz. The asterisk indicates the frequency band with the highest performance.

Figure 3A and B show the variance in predictive performance based on the window length of the resting-state EEG signal (average AUC of the four features at each window length is shown in Fig. 3A, the AUC of Feature group B at each window length is shown in Fig. 3B). As shown in Fig. 3A, the highest AUC of the four features at each window length was observed at 210 s (0.673 ± 0.076); this value was significantly different from that at other window lengths except 120, 150, 180, 240, and 270 s. As shown in Fig. 3B, the highest AUC of Feature group B at each window length was observed at 150 s (0.838 ± 0.102); this value was significantly different from that at almost all other window lengths. Detailed information regarding these analyses is shown in Supplementary Table 3. Figure 3C shows the AUC of each frequency band at the optimal EEG window length for Feature 2. The highest AUC in the low gamma band (frequency band of 39–41 Hz) was 0.748 ± 0.163, and the AUC showed a tendency to increase from the low-frequency band to the high-frequency band. In particular, it was found that the high beta (beta 3; AUC = 0.645 ± 0.050) and low gamma bands (AUC = 0.668 ± 0.057) showed better performance than the other frequency bands (delta: AUC = 0.522 ± 0.005, theta: AUC = 0.520 ± 0.100. alpha: AUC = 0.509 ± 0.131, beta1: AUC = 0.600 ± 0.047, beta2: AUC = 0.525 ± 0.085).

## Discussion

In this study, we showed the effects of using spectral and temporal filters on the prediction of outcomes among patients with TLE. Our main findings are as follows. (1) When predicting the outcome of TLE patients using resting-state EEG signals, simultaneously optimizing the spectral and temporal ranges greatly improved performance. (2) When the EEG window length is greater than 2 min, using the gamma band and statistical parameters (especially the kurtosis and the maximum value) as features had a substantial impact on the prediction performance.

### Synergy of spectral and temporal filters

We evaluated the use of each analysis strategy to compare and investigate the effect of spectral and temporal filters on prediction performance. When only one filter (spectral or temporal) was optimized, the performance was increased compared to the scenario in which no filer is optimized; however, the performance was further improved when both filters were optimized (Fig. 1). These results suggest that spectral and temporal filters must be used together to achieve a significant increase in performance, especially when using long-term EEG signals such as resting-state EEG. Additionally, we quantitatively compared the topology plot of each analysis strategy in terms of CS and ED, which represent the similarity of spatial patterns and the difference between the patterns, respectively. When the spectral filter was optimized, the CS between the two groups (SF and NSF) increased, which means that these groups had similar spatial patterns. The temporal filter seemed to be related to ED, indicating an increase in the intensity of the patterns; ED increased substantially when the spectral filter was applied. These results also show that synergy in the similarities of spatial patterns (CS and ED) occurs when the spectral and temporal filters are optimized simultaneously (Supplementary Table 2).

### Appropriate optimal EEG window length and EEG frequency band for SF prediction

As shown in Fig. 3A and B, we found that increasing the window length of the resting-state EEG signals to greater than 2 min led to a significant improvement in performance. We believe that the discriminative power of features could (1) exist in a specific section of the EEG signals or (2) occur over a specific length of EEG signals (or both). As resting-state EEG involves no specific stimulus or action^32^, it is expected that the discriminative power of features would occur over a specific length of time rather than in a specific section.

As shown in Fig. 3C, we compared the SF prediction performances within narrow frequency bands. The use of the low gamma band (30–50 Hz) with Feature 2 (statistical parameters) led to a higher predictive value.

Strengthening or weakening of cognitive function is one of the side effects of ASM treatment, which is secondary to the intended purpose of seizure control^33–35^. Although it is well known that long-term treatment with ASMs can adversely affect cognitive functions, such as attention, vigilance, and psychomotor speed^36,37^, some ASMs (e.g., carbamazepine, lamotrigine, valproate) have positive psychotropic effects^38^.

EEG modulation in the gamma band (> 30 Hz), has been shown to be correlated with large-scale brain network activity^39^. In particular, modulation in this band is known to play a crucial role in cognitive processes (e.g., working memory, attention, and perceptual grouping) and is thus assumed to reflect the consciousness level^40,41^.

Additionally, the EEG modulation in the gamma band was reflected in the kurtosis and the maximum value. One phenomenon—namely, sharp wave ripples—may account for the processes assessed by kurtosis and maximum values. Sharp wave ripples, which support the consolidation of recently acquired memories or the planning of future actions, consist of several spectral components: a slow sharp wave (5–15 Hz), a high-frequency “ripple” oscillation (150–200 Hz), and a slow “gamma” oscillation (20–40 Hz). The fusion of sharp wave ripples could also be reflected as increased power in the slow gamma band^42^. These prior findings lend credibility to our results with respect to the importance of low gamma and Feature 2.

### Limitations and future work

Our study has several limitations. First, our analysis was based on individual EEG segments for each patient. We only drew segments once for all window lengths because we were comparing performance across window lengths, and thus, we suspected that having different numbers of epochs for different window lengths might affect the results. The use of only one segment per patient might not fully capture the variability inherent in EEG signals. This approach may limit the generalizability of our findings, as multiple segments could provide a more comprehensive view of each patient's EEG characteristics. Second, the highest frequency bands that can be observed through scalp EEG signals are only approximately 50 Hz^43^. Therefore, it is necessary to investigate frequency ranges higher than 50 Hz through another modality through an additional method. Third, most patients were already taking ASMs at the time of the EEG study. Fourth, because the dataset used in this study consisted of patients receiving mono- or polytherapy, it is difficult to characterize the effect of a particular ASM on the EEG signal. Finally, this retrospective study was conducted using a limited dataset, which could introduce bias in the characteristics of epilepsy patients. Future research should apply more sophisticated methods that can aggregate multiple frequency bands and multiple time segments using resting-state EEG signals.

## Conclusion

This study shows that the application of spectral and temporal filters to resting-state EEG signals enhanced the prediction of long-term patient outcomes when the spectral and temporal filters were simultaneously optimized. In particular, an EEG window length of greater than 2 min and the gamma band substantially impacted the prediction performance. This optimization strategy can be applied for the early identification of patients with drug-resistant epilepsy, as they are potential candidates for nonpharmacologic intervention.

## Materials and methods

### Patients and data collection

We retrospectively analyzed the medical records and EEG data of patients with TLE who visited Seoul National University Hospital between 2014 and 2021. All included patients had experienced at least one clinical seizure and were confirmed as having TLE based on seizure semiology, EEG, and/or 3.0-Tesla magnetic resonance imaging throughout the follow-up period. All patients received ASM during the follow-up period. We included patients whose initial EEG data were obtained using the NicoletOne® EEG system (Natus, San Carlo, CA, USA). Demographic and clinical characteristics, including baseline and final seizure frequencies, were obtained through a retrospective review of medical records. A total of 46 patients with TLE were selected and divided into two groups according to the final outcome: the SF group (seizure-free for the last year of follow-up, n = 22) and the NSF group (at least one seizure in the last year of follow-up, n = 24). In our capacity as a tertiary referral hospital, we identified only one treatment-naïve patient with TLE, while all other patients were already using ASMs at the time of EEG study. None of the patients had undergone ketogenic diet therapy. This study was conducted in accordance with the Declaration of Helsinki. This study was approved by the Institutional Review Board of Seoul National University Hospital (IRB No. H-2109-005-1251), and the need for informed consent was waived by the Institutional Review Board of Seoul National University Hospital due to the retrospective nature of the study. Detailed patient information can be found in Table 2.

**Table 2:** Demographic and clinical characteristics of the seizure-free and non-seizure-free groups.

	Seizure-free (n = 22)	Non-seizure-free (n = 24)	p value	Effect size
Sex (N, %)			0.796^a^	0.038^b^
Male	12 (54.5%)	14 (58.3%)		
Female	10 (45.5%)	10 (41.7%)		
Age at EEG study (years, mean ± SD)	42.95 ± 18.09	39.29 ± 16.12	0.471^c^	0.214^d^
Onset age of epilepsy (years, mean ± SD)	27.50 ± 16.58	23.00 ± 12.13	0.296^c^	0.312^d^
Duration from onset to EEG (years, mean ± SD)	15.45 ± 13.27	16.29 ± 13.11	0.831^c^	0.064^d^
Follow-up duration (months, mean ± SD)	55.86 ± 18.85	46.83 ± 32.39	0.113^e^	0.234^f^
Seizure types (N, %)			0.603^a^	0.077^b^
Focal seizures only	8 (36.4%)	7 (29.2%)		
Focal & focal to bilateral seizures	14 (63.6%)	17 (70.8%)		
Etiology (N, %)			0.692^g^	0.201^b^
Hippocampal sclerosis	4 (18.2%)	7 (29.2%)		
Focal cortical dysplasia	0 (0.0%)	1 (4.2%)		
Trauma	1 (4.5%)	1 (4.2%)		
Unknown	17 (77.3%)	15 (62.5%)		
Epileptic focus (N, %)			0.281^g^	0.308^b^
Left	15 (68.2%)	13 (54.2%)		
Right	8 (33.3%)	6 (27.3%)		
Bilateral	3 (12.5%)	0 (0.0%)		
Unknown	0 (0.0%)	1 (4.5%)		
Number of ASMs used at the time of the EEG study (mean ± SD)	1.55 ± 1.01	2.42 ± 1.02	0.001^e^	0.476^f^
Seizure frequency at the time of the EEG study (per month, mean ± SD)	2.97 ± 12.75	3.33 ± 12.15	0.013^e^	0.367^f^
IED on initial EEG (N, %)	5 (22.7%)	11 (45.8%)	0.100^a^	0.242^b^
Family history of epilepsy (N, %)	3 (13.6%)	0 (0.0%)	0.101^g^	0.276^b^
History of febrile convulsion (N, %)	2 (9.1%)	0 (0.0%)	0.223^g^	0.223^b^
History of CNS infection (N, %)	0 (0.0%)	0 (0.0%)	NA	NA
History of head trauma (N, %)	3 (13.6%)	2 (8.3%)	0.659^g^	0.085^b^
History of epilepsy surgery (N, %)	1 (4.5%)	2 (8.3%)	1.000^g^	0.077^b^

EEG, electroencephalography; SD, standard deviation; IED, interictal epileptic discharge; ASM, antiseizure medication; CNS, central nervous system.

^a^Chi-square test.

^b^Cramer’s *V*.

^c^Student ‘s t test.

^d^Cohen’s *d*.

^e^Mann‒Whitney U test.

^f^Mann‒Whitney effect size *r*.

^g^Fisher’s exact test.

### EEG recording

All EEG data were acquired with the NicoletOne® EEG system using the modified international 10–20 electrode placement system (electrodes: Fp1, Fp2, T1, F7, F3, Fz, F4, F8, T2, T3, C3, Cz, C4, T4, T5, P3, Pz, P4, T6, O1, and O2), with a sampling rate of 250 Hz, a hardware high-pass filter of 0.1 Hz, and a hardware low-pass filter of 500 Hz. All electrode impedances were maintained below 10 kΩ. After photic stimulation and hyperventilation, the patients were asked to close their eyes to collect resting-state EEG data. The first 5 min of resting-state EEG data were processed for further analysis. Among the participants, 1 out of 22 SF patients (4.5%) and 4 out of 24 NSF patients (16.7%) exhibited interictal epileptiform discharges within the first 5 min of EEG recordings (p = 0.349).

### Preprocessing

The resting-state EEG signals, recorded from 20 to 320 s, were epoched and then scaled by 10^6^ to convert the measurements from volts to microvolts (µV), thus enhancing both the relevance and clarity of the data^44^. Data from all 21 channels were used in further analysis. However, data referencing was conducted only with the following EEG channels: F3, Fz, F4, C3, Cz, C4, P3, Pz, P4, O1, and O2^45^.

### Analysis strategy

**Figure 4:**
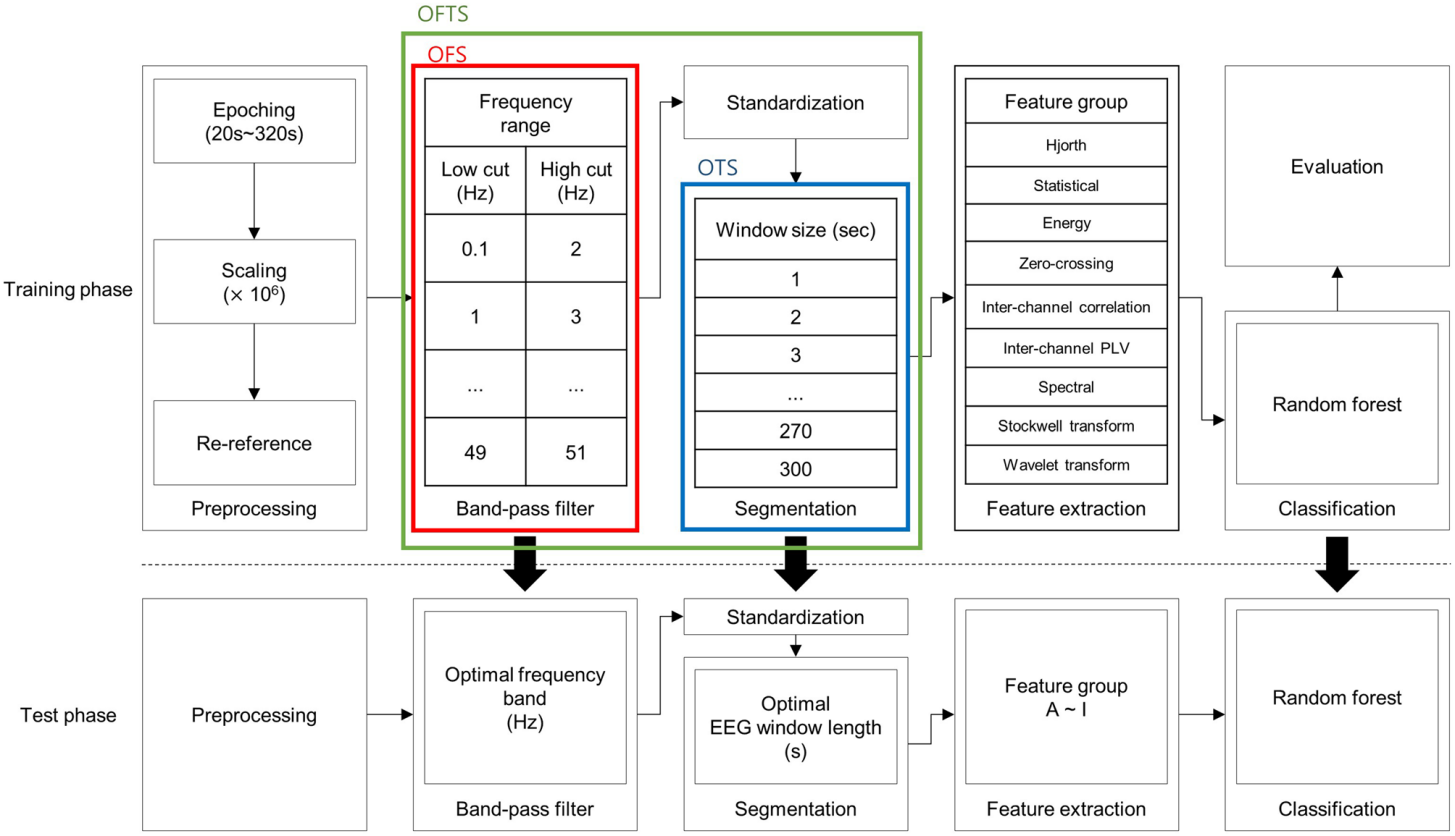
Analysis strategy for determining the optimal spectral and temporal range.

To determine the effect of appropriate spectral and temporal ranges in predicting SF outcomes of TLE patients, the following three strategies were compared. (1) In the OTS strategy, a grid search was applied to optimize the temporal range of the EEG signals in a fixed frequency band (0.1–51 Hz). The temporal segments were extracted only once with different durations. All segments started precisely at the 0-s mark of the resting-state EEG data. The target temporal range was set at 1-s intervals from 1 to 30 s and 30-s intervals from 30 to 300 s (thus yielding a total of 39 temporal segments, 1, 2, 3, …, 27, 28, 29, 30, 60, …, 90, 120, 150, …, 300 s). (2) In the OFS strategy, a grid search was applied to optimize the spectral range of EEG signals from 0.1 Hz (low cut) to 2 Hz (high cut) up to 49 Hz (low cut) to 51 Hz (high cut) (a total of 50 bands spanning 2 Hz) for the total resting state (300 s). (3) In the OFTS strategy, a two-grid search was applied to 50 spectral and 39 temporal ranges to optimize the spectral and temporal ranges simultaneously. Then, bandpass filtering, standardization^46^ and segmentation were sequentially performed. Figure 4 shows the analysis pipeline for OFTS. In the case of NS, total resting state (300 s) and fixed-frequency bandpass cutoff from 0.1 Hz (low cut) to 51 Hz (high cut) were applied.

### Feature extraction

Among the many EEG features, four types of features were selected based on the following three questions (1) Is it a time-domain feature? (The analysis pipeline included a narrow bandpass filter). (2) Has it ever been used in an EEG-based epilepsy study? (3) What is the computational cost? (Supplementary Table 4). The selected features were as follows: Hjorth parameters (Feature group A), statistical parameters (Feature group B), energy (Feature group C), zero-crossing rate (Feature group D), interchannel correlation (ICC) (Feature group E), interchannel phase locking value (ICPLV) (Feature group F), spectral power (Feature group G), Stockwell transform (ST) (Feature group H), and wavelet transform (WT) (Feature group I).

### Hjorth parameters (Feature group A)

Hjorth parameters consist of activity, mobility, and complexity, which represent the signal power, mean frequency, and change in frequency, respectively^47^. In many epilepsy studies, these parameters have been used to detect and diagnose seizures^48,49^ or to predict seizure recurrence after ASM withdrawal^50^. A total of 63 Hjorth parameters were acquired using Eqs. (1)–(3) and used as ML inputs.

1 $$ {\text{Activity}}\left( {\text{X}} \right) = {\text{var}}\left( {{\text{X}}\left( {\text{t}} \right)} \right) $$

2 $$ {\text{Mobility}} = \frac{{\sqrt {{\text{var}}\left( {{{{\text X}^{\prime}}}\left( {\text{t}} \right)} \right)} }}{{\sqrt {{\text{var}}\left( {{\text{X}}\left( {\text{t}} \right)} \right)} }} $$

3 $$ {\text{Complexity}} = \frac{{\sqrt {{\text{var}}\left( {{{{\text X}^{\prime\prime}}}\left( {\text{t}} \right)} \right)} /\sqrt {{\text{var}}\left( {{{{\text X}^{\prime}}}\left( {\text{t}} \right)} \right)} }}{{\sqrt {{\text{var}}\left( {{{{\text X}^{\prime}}}\left( {\text{t}} \right)} \right)} /\sqrt {{\text{var}}\left( {{\text{X}}\left( {\text{t}} \right)} \right)} }} $$

### Statistical parameters (Feature group B)

In many EEG-based epilepsy studies, statistical parameters have been used as features to detect seizures^51^, distinguish healthy participants from epilepsy patients^52^ and predict LEV treatment responses^17,51^. We selected six of the most commonly used statistical indicators (i.e., skewness, kurtosis, and the mean, median, minimum, and maximum values) as Feature group B. Variance, which is one of the most commonly used statistical indicators, was excluded from Feature group B because it was included in Feature group A (Hjorth parameters). A total of 126 statistical parameters were acquired using Eqs. (4)–(9) and used as ML inputs.

4 $$ {\text{Skewness}}\left( {\text{X}} \right) = {\text{E}}\left[ {\left( {\frac{X\left( t \right) - \mu }{\sigma }} \right)^{3} } \right], $$

5 $$ {\text{Kurtosis}}\left( {\text{X}} \right) = {\text{E}}\left[ {\left( {\frac{X\left( t \right) - \mu }{\sigma }} \right)^{4} } \right] $$

6 $$ {\text{Mean}}\left( {\text{X}} \right) = \frac{1}{n}\mathop \sum \limits_{t = 1}^{n} X\left( t \right) $$

7 $$ {\text{Median}}\left( {\text{X}} \right) = \left\{ {\begin{array}{*{20}l} {X\left( {\left( {t + 1} \right)/2} \right)} \hfill & { if\; t\; is\; odd} \hfill \\ {\left( {X\left( \frac{t}{2} \right) + X\left( {\frac{t}{2} + 1} \right)} \right)/2} \hfill & {if\; t\; is\; even} \hfill \\ \end{array} } \right. $$

8 $$ {\text{Min}}\left( {\text{X}} \right) = X\left( {t_{0} } \right),\;\; if\left( {\forall {\text{t}} \in {\text{T}}} \right) X\left( {t_{0} } \right) \le X\left( t \right) $$

9 $$ {\text{Max}}\left( {\text{X}} \right) = X\left( {t_{0} } \right),\;\; if\left( {\forall {\text{t}} \in {\text{T}}} \right) X\left( {t_{0} } \right) \ge X\left( t \right) $$

### Energy (Feature group C)

Many studies have used energy as an indicator of brain activity^18,19^. Therefore, the linear and nonlinear energy of the EEG signals were included in Feature group C^52^. A total of 42 energy parameters were acquired using Eqs. (10), (11) and used as ML inputs.

10 $$ Energy_{Linear} \left( {\text{X}} \right) = \frac{1}{n}\mathop \sum \limits_{t = 1}^{n} X\left( t \right)*X\left( t \right) $$

11 $$ Energy_{Non - linear} \left( {\text{X}} \right) = \frac{1}{n - 2}\left( {\mathop \sum \limits_{t = 2}^{n - 1} X\left( t \right)*X\left( t \right) - \mathop \sum \limits_{t = 3}^{n} X\left( t \right)*X\left( t \right)} \right) $$

### Zero-crossing rate (Feature group D)

The zero-crossing rate is the rate at which a signal changes from positive to zero to negative or from negative to zero to positive^53^. This parameter has been used to detect or classify seizures from normal EEG signals in many studies^54,55^. In this study, the zero-crossing rate and its first derivative were included in Feature group D^52^. A total of 42 zero-crossing parameters were acquired using Eqs. (12), (13) and used as ML inputs.

12 $$ {\text{Zero}} - {\text{crossing rate }}\left( {\text{X}} \right) = \frac{1}{2}\mathop \sum \limits_{t = 2}^{n} \left| {sign(X\left( t \right) - sign(X\left( {t - 1} \right)} \right| $$

13 $$ {\text{Zero}} - {\text{crossing}}_{derivative} \left( {\text{X}} \right) = \frac{1}{2}\mathop \sum \limits_{t = 2}^{n} \left| {sign(X^{\prime}\left( t \right) - sign(X^{\prime}\left( {t - 1} \right)} \right| $$

### ICC (Feature group E)

In the context of EEG analysis, cross-correlation is a powerful tool^56,57^ that offers unique insights into the functional connectivity and relationships between different brain regions. We used the Pearson correlation coefficient as the correlation coefficient in Eq. (14), which is a measure of the linear correlation between two variables X and Y. A total of 210 ICC were acquired using Eq. (14) and 20 graph measurements^23,58^ were used as ML inputs. Graph measurements were performed using NetworkX^59^ and nilearn^60^ Python libraries.

14 $$ {\text{Inter}} - {\text{channel}}_{correalation} \left( {{\text{X}},{\text{Y}}} \right) = \frac{{\mathop \sum \nolimits_{t = 1}^{T} \left( {X\left( t \right) - \overline{X}} \right)\left( {Y\left( t \right) - \overline{Y}} \right)}}{{\sqrt {\mathop \sum \nolimits_{t = 1}^{T} \left( {X\left( t \right) - \overline{X}} \right)^{2} \left( {Y\left( t \right) - \overline{Y}} \right)^{2} } }} $$

### ICPLV (Feature group F)

In the context of EEG analysis, ICPLV is a powerful tool for investigating phase synchronization between brain regions. Its ability to provide insights into the timing and coordination of brain activities makes it invaluable in both research and clinical settings^61,62^. A total of 210 ICPLVs were acquired using Eq. (15), and 20 graph measurements^23,58^ were used as ML inputs.

15 $$ {\text{Inter}} - {\text{channel}}_{plv} \left( {{\text{X}},{\text{Y}}} \right) = \left| {\frac{1}{{\text{T}}}\mathop \sum \limits_{t = 1}^{T} e^{{i\left( {\emptyset_{X\left( t \right)} - \emptyset_{Y\left( t \right)} } \right)}} } \right| $$

### Spectral parameters (Feature group G)

Spectral power and phase, which are acquired using fast Fourier transform (FFT) (Eq. 16), are crucial components of EEG signal analysis, These parameters provide deep insights into brain function and neural activity.^63,64^ We extracted five spectral parameters: mean, median, minimum, maximum, skewness and standard deviation of power and phase. A total of 126 spectral parameters were acquired using Eq. (16) and used as ML inputs.

16 $$ Spectral - power\left( {\text{X}} \right) = \mathop \sum \limits_{t = 1}^{T - 1} X\left( t \right)*e^{{\frac{i2\pi }{T}kt}} $$

### ST parameters (Feature group H)

The ST is a linear transform that combines elements of the wavelet transform and the Fourier transform. It provides a time–frequency representation of a signal, similar to the wavelet transform, but it exhibits properties more akin to the Fourier transform^65,66^. We extracted five ST parameters mean, median, minimum, maximum, skewness and standard deviation. The *stockwell* python package was used to estimate ST. A total of 126 ST parameters were used as ML inputs.

### WT (Feature group I)

Wavelet coefficients derives from the application of a wavelet transform to a signal. The wavelet transform decomposes the signal into components that vary in both time and frequency, unlike the Fourier transform, which only provides frequency information^67,68^. The wavelet coefficient of the continuous wavelet transform was acquired using the pywt.cwt function in Python (‘wavelet = db4’)^69^. We extracted five wavelet coefficient parameters: mean, median, minimum, maximum, skewness and standard deviation of square of absolute values of WT. A total of 126 WT parameters were used as ML inputs.

### Classification and statistical analysis

The classification was performed using the RF model^26^ in the main experiment, and LDA^27^, XGB^28^, and CATB^29^ were used for the additional experiment. We treated each EEG channel as a separate input and only used a single segment of the temporal range. The area under the receiver operating characteristic curve, accuracy, F1 score, true positive rate, true negative rate, positive predictive value, and negative predictive value for each feature were compared through five-fold nested cross-validation with grid search, in which the number of patients in the test dataset was 9 or 10. The hyperparameter of each fold of each model was set based on grid-search (Supplementary Table 5). The Mann‒Whitney U test and Cliff’s delta were used for all statistical analyses, including the comparison of each feature’s performance.

### Conflict of interest/ethical publication statement

None of the authors has any conflict of interest to disclose. We confirm that we have read the Journal’s position on issues involved in ethical publication and affirm that this report is consistent with those guidelines. This study was approved by the Institutional Review Board of Seoul National University Hospital (IRB No. H-2102-178-1200), and the need for informed consent was waived because of the retrospective design.

### Supplementary Information

Supplementary Tables.

## Data Availability

The datasets used and/or analyzed during the current study are available from the corresponding author on reasonable request.

## References

[1] Fisher RS (2014). ILAE official report: A practical clinical definition of epilepsy. Epilepsia.

[2] Fisher RS (2005). Epileptic seizures and epilepsy: Definitions proposed by the International League Against Epilepsy (ILAE) and the International Bureau for Epilepsy (IBE). Epilepsia.

[3] Chang BS, Lowenstein DH (2003). Epilepsy. N. Engl. J. Med..

[4] Annegers JF, Rocca WA, Hauser WA (1996). Causes of epilepsy: Contributions of the Rochester epidemiology project. Mayo Clin. Proc..

[5] Shorvon SD (2011). The causes of epilepsy: Changing concepts of etiology of epilepsy over the past 150 years. Epilepsia.

[6] Scheffer IE (2017). ILAE classification of the epilepsies: Position paper of the ILAE Commission for Classification and Terminology. Epilepsia.

[7] Tanaka T (2021). Antiseizure medications for post-stroke epilepsy: A real-world prospective cohort study. Brain Behav..

[8] Löscher W, Klein P (2021). The pharmacology and clinical efficacy of antiseizure medications: From bromide salts to cenobamate and beyond. CNS Drugs.

[9] Sillanpää M, Schmidt D (2017). Long-term outcome of medically treated epilepsy. Seizure.

[10] Sillanpää M (2000). Long-term outcome of epilepsy. Epileptic Disord..

[11] Tang F, Hartz AMS, Bauer B (2017). Drug-resistant epilepsy: Multiple hypotheses, few answers. Front. Neurol..

[12] Kwan P (2010). Definition of drug resistant epilepsy: Consensus proposal by the ad hoc Task Force of the ILAE Commission on Therapeutic Strategies. Epilepsia.

[13] Xiao F (2021). Effect of anti-seizure medications on functional anatomy of language: A perspective from language functional magnetic resonance imaging. Front. Neurosci..

[14] Britton, J. W. *et al.* In *Electroencephalography (EEG): An Introductory Text and Atlas of Normal and Abnormal Findings in Adults, Children, and Infants* (eds St. Louis, E. K. & Frey, L. C.) (American Epilepsy Society Copyright ©2016 by American Epilepsy Society, 2016). 27748095

[15] Pantaleon L (2019). Why measuring outcomes is important in health care. J. Vet. Intern. Med..

[16] Zhang JH (2018). Personalized prediction model for seizure-free epilepsy with levetiracetam therapy: A retrospective data analysis using support vector machine. Br. J. Clin. Pharmacol..

[17] Croce P (2021). Machine learning for predicting levetiracetam treatment response in temporal lobe epilepsy. Clin. Neurophysiol..

[18] Ricci L (2021). Measuring the effects of first antiepileptic medication in Temporal Lobe Epilepsy: Predictive value of quantitative-EEG analysis. Clin. Neurophysiol..

[19] Lanzone J (2021). The effect of Perampanel on EEG spectral power and connectivity in patients with focal epilepsy. Clin. Neurophysiol..

[20] Jobert M (2012). Guidelines for the recording and evaluation of pharmaco-EEG data in man: The International Pharmaco-EEG Society (IPEG). Neuropsychobiology.

[21] Kai Keng, A., Zheng Yang, C., Haihong, Z. & Cuntai, G. In *2008 IEEE International Joint Conference on Neural Networks (IEEE World Congress on Computational Intelligence)* 2390–2397 (2008).

[22] Lee SB (2022). Predicting Parkinson's disease using gradient boosting decision tree models with electroencephalography signals. Parkinson. Relat. Disord..

[23] Thangavel P (2022). Improving automated diagnosis of epilepsy from EEGs beyond IEDs. J. Neural Eng..

[24] Christou V (2022). Evaluating the window size’s role in automatic EEG epilepsy detection. Sensors.

[25] Kang KW, Lee H, Shin JY, Moon HJ, Lee SY (2022). Trends in prescribing of antiseizure medications in South Korea: Real-world evidence for treated patients with epilepsy. J. Clin. Neurol..

[26] Tin Kam, H. In *Proceedings of 3rd International Conference on Document Analysis and Recognition, vol. 271 *278–282 (2020).

[27] McLachlan, G. J. *Discriminant Analysis and Statistical Pattern Recognition* (John Wiley & Sons, 2005).

[28] Chen, T. & Guestrin, C. In *Proceedings of the 22nd ACM SIGKDD International Conference on Knowledge Discovery and Data Mining* 785–794 (Association for Computing Machinery, 2016).

[29] Prokhorenkova, L., Gusev, G., Vorobev, A., Dorogush, A. V. & Gulin, A. CatBoost: unbiased boosting with categorical features. *Advances in neural information processing systems* **31** (2018).

[30] Ceri, S. *et al. Morgan Kaufmann Series in Data Management Systems: Designing Data-Intensive Web Applications* (Morgan Kaufmann, 2003).

[31] Smith, K. J. *Precalculus: A Functional Approach to Graphing And Problem Solving* (Jones & Bartlett Publishers, 2011).

[32] Bai Y, Xia X, Li X (2017). A review of resting-state electroencephalography analysis in disorders of consciousness. Front. Neurol..

[33] Saletu B, Grünberger J (1984). Early clinical pharmacological trials with a new anti-epileptic, milacemide, using pharmaco-EEG and psychometry. Methods Find. Exp. Clin. Pharmacol..

[34] Saletu B, Grünberger J, Linzmayer L (1986). Evaluation of encephalotropic and psychotropic properties of gabapentin in man by pharmaco-EEG and psychometry. Int. J. Clin. Pharmacol. Ther. Toxicol..

[35] Salinsky MC, Oken BS, Storzbach D, Dodrill CB (2003). Assessment of CNS effects of antiepileptic drugs by using quantitative EEG measures. Epilepsia.

[36] Park SP, Kwon SH (2008). Cognitive effects of antiepileptic drugs. J. Clin. Neurol..

[37] Eddy CM, Rickards HE, Cavanna AE (2011). The cognitive impact of antiepileptic drugs. Ther. Adv. Neurol. Disord..

[38] Cavanna AE, Ali F, Rickards HE, McCorry D (2010). Behavioral and cognitive effects of anti-epileptic drugs. Discov. Med..

[39] McDermott B (2018). Gamma band neural stimulation in humans and the promise of a new modality to prevent and treat alzheimer's disease. J. Alzheim. Dis..

[40] Herrmann CS, Demiralp T (2005). Human EEG gamma oscillations in neuropsychiatric disorders. Clin. Neurophysiol..

[41] Engel AK, Fries P, Singer W (2001). Dynamic predictions: Oscillations and synchrony in top-down processing. Nat. Rev. Neurosci..

[42] Oliva A, Fernández-Ruiz A, Fermino-de-Oliveira E, Buzsáki G (2018). Origin of gamma frequency power during hippocampal sharp-wave ripples. Cell Rep..

[43] Motamedi-Fakhr S, Moshrefi-Torbati M, Hill M, Hill CM, White PR (2014). Signal processing techniques applied to human sleep EEG signals—a review. Biomed.l Signal Process. Control.

[44] Gramfort A (2014). MNE software for processing MEG and EEG data. Neuroimage.

[45] Yao D (2019). Which reference should we use for EEG and ERP practice?. Brain Topogr..

[46] Schirrmeister RT (2017). Deep learning with convolutional neural networks for EEG decoding and visualization. Hum. Brain Mapping.

[47] Hjorth B (1970). EEG analysis based on time domain properties. Electroencephalogr. Clin. Neurophysiol..

[48] Päivinen N (2005). Epileptic seizure detection: A nonlinear viewpoint. Comput. Methods Programs Biomed..

[49] Tanveer, M., Pachori, R. B. & Angami, N. V. In *2018 IEEE Symposium Series on Computational Intelligence (SSCI)* 2180–2185 (2018).

[50] Ouyang CS, Yang RC, Wu RC, Chiang CT, Lin LC (2020). Determination of antiepileptic drugs withdrawal through EEG Hjorth parameter analysis. Int. J. Neural Syst..

[51] Zhang S (2022). A combination of statistical parameters for epileptic seizure detection and classification using VMD and NLTWSVM. Biocybernet. Biomed. Eng..

[52] Gemein LAW (2020). Machine-learning-based diagnostics of EEG pathology. Neuroimage.

[53] Chen CH (1988). Signal Processing Handbook.

[54] Pyrzowski J (2021). Zero-crossing patterns reveal subtle epileptiform discharges in the scalp EEG. Sci. Rep..

[55] Shahidi-Zandi A, Tafreshi R, Javidan M, Dumont GA (2010). Predicting temporal lobe epileptic seizures based on zero-crossing interval analysis in scalp EEG. Annu. Int. Conf. IEEE Eng. Med. Biol. Soc..

[56] Chandaka S, Chatterjee A, Munshi S (2009). Cross-correlation aided support vector machine classifier for classification of EEG signals. Expert Syst. Appl..

[57] Abdullah H, Maddage NC, Cosic I, Cvetkovic D (2010). Cross-correlation of EEG frequency bands and heart rate variability for sleep apnoea classification. Med. Biol. Eng. Comput..

[58] Fagerholm ED, Hellyer PJ, Scott G, Leech R, Sharp DJ (2015). Disconnection of network hubs and cognitive impairment after traumatic brain injury. Brain.

[59] Hagberg, A., Swart, P. & S Chult, D. *Exploring Network Structure, Dynamics, and Function Using NetworkX* (Los Alamos National Lab.(LANL), 2008).

[60] Abraham A (2014). Machine learning for neuroimaging with scikit-learn. Front. Neuroinform..

[61] Jian W, Chen M, McFarland DJ (2017). EEG based zero-phase phase-locking value (PLV) and effects of spatial filtering during actual movement. Brain Res. Bull..

[62] di Biase L (2023). Quantitative high density EEG brain connectivity evaluation in parkinson’s disease: The phase locking value (PLV). J. Clin. Med..

[63] Shakshi RJ, Jaswal R (2016). Brain wave classification and feature extraction of EEG signal by using FFT on lab view. Int. Res. J. Eng. Technol..

[64] Murugappan, M., Murugappan, S. & Gerard, C. In *2014 IEEE 10th International Colloquium on Signal Processing and its Applications.* 25–30 (IEEE, 2014).

[65] Stockwell RG, Mansinha L, Lowe R (1996). Localization of the complex spectrum: The S transform. IEEE Trans. Signal Process..

[66] Kalbkhani H, Shayesteh MG (2017). Stockwell transform for epileptic seizure detection from EEG signals. Biomed. Signal Process. Control.

[67] Li M, Chen W, Zhang T (2017). Automatic epileptic EEG detection using DT-CWT-based non-linear features. Biomed. Signal Process. Control.

[68] Kumar JLM (2021). The classification of EEG-based wink signals: A CWT-transfer learning pipeline. ICT Express.

[69] Lee G, Gommers R, Waselewski F, Wohlfahrt K, O'Leary A (2019). PyWavelets: A Python package for wavelet analysis. J. Open Sourc. Softw..

