# Supplementary material for: Using spectral and temporal filters with EEG signal to predict the temporal lobe epilepsy outcome after antiseizure medication via machine learning
Source: Sci Rep. 2023 Dec 18;13:22532. doi: 10.1038/s41598-023-49255-2 (PMC10728218; doi:10.1038/s41598-023-49255-2)
Supplement: Supplementary file 1 — Supplementary Tables. [file 41598_2023_49255_MOESM1_ESM.docx]

**Supplementary Table 1.** Performance of Feature 2 on the other machine learning classifiers.

|  | AUC | ACC | F1 score | TPR | TNR | PPV | NPV |
| --- | --- | --- | --- | --- | --- | --- | --- |
| CATB | 0.728±0.135 | 0.736±0.168 | 0.685±0.230 | 0.660±0.260 | 0.790±0.128 | 0.727±0.224 | 0.750±0.167 |
| LDA | 0.748±0.186 | 0.802±0.149 | 0.732±0.231 | 0.700±0.292 | **0.880±0.098** | 0.817±0.186 | 0.817±0.166 |
| RF | 0.748±0.163 | 0.824±0.135 | 0.767±0.213 | 0.750±0.274 | 0.880±0.160 | **0.887±0.157** | **0.842±0.145** |
| XGB | **0.765±0.179** | **0.827±0.112** | **0.798±0.126** | **0.760±0.159** | 0.880±0.160 | 0.880±0.160 | 0.820±0.099 |

CATB: categorical boosting, LDA: linear discriminant analysis, RF: random forest, XGB: extreme gradient boosting.

**Supplementary Table 2.** Cosine similarity and Euclidean distance of each analysis strategy between the seizure-free and nonseizure-free groups. NS: no strategy, OTS: optimal time strategy, OFS: optimal frequency strategy, OFTS: optimal frequency and time strategy.

|  | Kurtosis | | Maximum value | |
| --- | --- | --- | --- | --- |
|  | Cosine similarity (CS) | Euclidean distance (ED) | Cosine similarity (CS) | Euclidean distance (ED) |
| NS | 0.803 | 3.015 | 0.995 | 2.680 |
| OTS | 0.816 | 3.357 | 0.996 | 2.388 |
| OFS | 0.972 | 2.992 | 0.998 | 4.879 |
| OFTS | 0.936 | 4.929 | 0.998 | 5.935 |

NS: no strategy, OTS: optimal temporal selection, OFS: optimal frequency selection, OFTS: optimal frequency and temporal selection

**Supplementary Table 3.** Statistical comparison according to length of window segment

|  | Average | | Feature group B | |
| --- | --- | --- | --- | --- |
| Segment length [sec] | p-value | Cliff d | p-value | Cliff d |
| 1 | >0.001 | 0.491 | 0.016 | 0.920 |
| 2 | >0.001 | 0.707 | 0.056 | 0.760 |
| 3 | >0.001 | 0.432 | 0.032 | 0.840 |
| 4 | >0.001 | 0.493 | 0.016 | 0.920 |
| 5 | >0.001 | 0.442 | 0.016 | 0.920 |
| 6 | 0.001 | 0.423 | 0.095 | 0.680 |
| 7 | >0.001 | 0.518 | 0.056 | 0.760 |
| 8 | >0.001 | 0.618 | 0.016 | 0.920 |
| 9 | >0.001 | 0.520 | 0.016 | 0.920 |
| 10 | 0.003 | 0.365 | 0.032 | 0.840 |
| 11 | 0.001 | 0.408 | 0.056 | 0.760 |
| 12 | >0.001 | 0.812 | 0.008 | 1.000 |
| 13 | 0.022 | 0.280 | 0.016 | 0.920 |
| 14 | 0.005 | 0.344 | 0.016 | 0.920 |
| 15 | 0.001 | 0.395 | 0.095 | 0.680 |
| 16 | >0.001 | 0.459 | 0.056 | 0.760 |
| 17 | >0.001 | 0.513 | 0.056 | 0.760 |
| 18 | 0.010 | 0.315 | 0.032 | 0.840 |
| 19 | 0.003 | 0.361 | 0.056 | 0.760 |
| 20 | 0.002 | 0.373 | 0.151 | 0.600 |
| 21 | >0.001 | 0.473 | 0.016 | 0.920 |
| 22 | 0.002 | 0.381 | 0.056 | 0.760 |
| 23 | 0.002 | 0.384 | 0.056 | 0.760 |
| 24 | >0.001 | 0.520 | 0.056 | 0.760 |
| 25 | 0.009 | 0.320 | 0.095 | 0.680 |
| 26 | >0.001 | 0.436 | 0.016 | 0.920 |
| 27 | 0.039 | 0.253 | 0.151 | 0.600 |
| 28 | >0.001 | 0.487 | 0.095 | 0.680 |
| 29 | 0.007 | 0.332 | 0.095 | 0.680 |
| 30 | 0.006 | 0.338 | 0.037 | 0.840 |
| 60 | 0.001 | 0.414 | 0.013 | 0.920 |
| 90 | 0.012 | 0.307 | 0.018 | 0.600 |
| 120 | 0.228 | 0.148 | 0.421 | 0.360 |
| 150 | 0.165 | 0.170 | Reference | |
| 180 | 0.693 | 0.049 | 0.548 | 0.280 |
| 210 | Reference | | 0.690 | 0.200 |
| 240 | 0.107 | 0.198 | 0.690 | 0.200 |
| 270 | 0.381 | 0.108 | 0.548 | 0.280 |
| 300 | 0.004 | 0.354 | 0.030 | 0.760 |

**Supplementary Table 4.** Computation cost of each model for 300 sec window length data

| Feature | Time [sec] |
| --- | --- |
| Hjorth | 0.284 |
| Statistic | 0.733 |
| Energy | 0.122 |
| Zero crossing | 0.240 |
| ICC | 7.004 |
| ICPLV | 61.068 |
| Spectral | 1.878 |
| ST | 215.698 |
| WT | 256.386 |

ICC: inter-channel correlation, ICPLV: inter-channel phase locking value, DFT: discrete Fourier transform, ST: Stockwell transform, WT: wavelet transform.

**Supplementary Table 5.** The hyperparameter grid of each model

| Model | Grid |
| --- | --- |
| CATB | Learning rate: [0.01, 0.05, 0.1] |
|  | Depth: [4, 6, 10] |
|  | L2 leaf regularization: [1, 3, 5] |
|  | Iterations: [500, 1000, 1500] |
| LDA | Solver: ['svd', 'lsqr', 'eigen'] |
|  | Shrinkage: [None, 'auto', 0.0-1.0 (in steps)] |
| RF | Number of trees: [100, 200, 500] |
|  | Max depth: [10, 20, 30] |
|  | Min samples split: [2, 5, 10] |
|  | Min samples leaf: [1, 2, 4] |
| XGB | Learning rate: [0.01, 0.1, 0.2] |
|  | Max depth: [3, 6, 10] |
|  | Subsample: [0.5, 0.75, 1] |
|  | N_estimators: [100, 200, 300] |

CATB: categorical boosting, LDA: linear discriminant analysis, RF: random forest, XGB: extreme gradient boosting.
